# Advances in Photonic Metasurfaces and Metastructures

**DOI:** 10.3390/nano15030153

**Published:** 2025-01-21

**Authors:** Viktoriia E. Babicheva

**Affiliations:** Department of Electrical and Computer Engineering, University of New Mexico, MSC01 1100, Albuquerque, NM 87131, USA; vbb@unm.edu

Photonic metasurfaces and metastructures have revolutionized the manipulation of electromagnetic waves across diverse applications, from optical communication to sensing and stealth technology. This *Editorial* highlights several advances in metasurface research, as covered in the Special Issue “Advances in Photonic Metasurfaces and Metastructures”. The generation of bi-directional full-color structural devices is discussed in the context of plasmonic metasurfaces, where the incidence polarization angle enables dynamic color control for applications such as anti-counterfeiting and optical information storage. Quarter-wave-plate meta-atom metasurfaces are explored for multi-channel vortex beam generation, leveraging concentric circular arrangements to independently modulate co- and cross-polarized components for high-capacity communications and particle manipulations. Additionally, van der Waals nanoantennas, leveraging hyperbolic materials such as hexagonal boron nitride, enable strong localized resonances and directional scattering, advancing metasurface functionality for compact photonic systems. An integrated fiber metasurface device in the mid-infrared band can achieve high-quality factors, sensitivity, and figures of merit when bilayer structures are introduced, supporting advances in tunable filters and sensing. A vanadium-dioxide-based dynamic coding metasurface is introduced for terahertz applications, demonstrating dual-band, wide-angle radar cross-section reduction with reconfigurable functionality through state transitions in vanadium dioxide. Microwave absorber metasurfaces employing flexible materials, such as polyethylene terephthalate and polyvinyl chloride, enable wideband absorptivity and angular stability for stealth applications. Together, these developments illustrate the potential of photonic metasurfaces to drive innovation in compact, multifunctional optical devices across the electromagnetic spectrum.

Structural color generation, achieved through nanostructured materials, has revolutionized how light is manipulated for vivid, tunable coloration. Unlike pigment-based methods, structural colors arise from interference, diffraction, or resonance of light, offering unparalleled durability and environmental stability. Advances in metasurface design have enabled dynamic control over color, making them ideal for applications in display technologies, anti-counterfeiting measures, and optical data storage. By integrating polarization-sensitive designs, these metasurfaces can encode multiple color channels or information streams within a single structure, enhancing functionality. This approach paves the way for compact, multifunctional optical devices with tailored spectral responses.

Bi-directional full-color generation from a plasmonic metasurface has numerically been demonstrated by adjusting the polarization angle of the incident light [1]. A three-module metasurface was engineered to exhibit polarization-angle-dependent optical responses, enabling the generation of various colors. By fine-tuning the metasurface dimensions, full-color output was shown to be achieved simultaneously in both reflection and transmission. The reflected color follows an additive model, while the transmitted color adheres to a subtractive model. Additionally, metasurface designs with different module configurations within each unit cell and variations in module combinations that alter the optical response have been explored. The findings indicate that the presence of specific modules can be detected by the perceived color under linearly polarized white light aligned with the module’s long axis. Leveraging polarization-tunable behavior, metasurfaces that encode three-channel information were designed and tested using two examples: images of three animals and data from three QR codes. The animal images were successfully reconstructed, and the QR codes were decoded to reveal their embedded content, enhancing the information capacity of the device. The proposed plasmonic structural color device demonstrates significant potential for applications in kaleidoscope generation, anti-counterfeiting measures, dynamic color displays, and optical data encoding.

Vortex beams, characterized by their helical phase fronts and orbital angular momentum (OAM), have garnered significant attention for their potential in optical communication, particle manipulation, and imaging. These beams provide unique advantages, such as the ability to carry multiple data channels through distinct OAM modes, enhancing bandwidth and efficiency. Recent advances in metasurfaces, particularly those employing geometric and propagation phase engineering, have enabled precise control over vortex beam generation and manipulation. By integrating compact and versatile meta-atoms, metasurfaces can produce complex beam patterns and polarization-dependent functionalities. This progress is driving innovation in fields that require miniaturized and multifunctional optical components.

Quarter-wave-plate (QWP) meta-atom metasurfaces have been reported to produce multi-channel vortex beams [2]. These metasurfaces were constructed by arranging QWP meta-atoms in systematically designed concentric circular rings. Leveraging the propagation phase and geometric phase within the QWP meta-atoms, the metasurfaces demonstrated the capability to independently control the co-polarized and cross-polarized components, resulting in the formation of a stationary co-polarized vortex and a deflected cross-polarized vortex. The deflection direction was determined by the polarization states of the incident light, attributed to the inherent chirality of the geometric phase. Theoretical analysis and numerical simulations validated the effectiveness of the proposed QWP meta-atom metasurfaces for controlling vortex fields and generating multi-channel beams. This work introduces wave-plate metasurfaces suitable for applications in high-capacity optical communications and multi-particle manipulation, emphasizing their importance in advancing the miniaturization and integration of optical systems.

The optical nanoantenna composed of a hyperbolic medium has been demonstrated to sustain intense localized resonant modes originating from propagating high-wavenumber waves in the hyperbolic medium [3]. The metasurface can be constructed using optical nanoantennas fabricated from the van der Waals material hexagonal boron nitride. A theoretical framework was employed to identify the mode structure and nature of resonances in cuboid nanoantennas. An electric quadrupole mode was shown to correspond to a resonant magnetic behavior in the nanoantenna, mirroring the excitation of magnetic resonances in nanoantennas with a high refractive index. The theoretical model effectively predicts the modes of cuboid nanoantennas due to the pronounced boundary reflections of high-wavenumber waves, a capability not applicable to plasmonic or high-refractive-index nanoantennas, where partial reflections and mode leakage from the cavity complicate the interpretation. In this metasurface, the activation of multipolar resonant modes leads to directional scattering and a reduction in the metasurface reflectance to zero, manifesting as the resonant Kerker phenomenon. Van der Waals nanoantennas can enable localized resonances and emerge as critical functional components for metasurfaces and multidimensional photonic systems. By designing highly efficient subwavelength scatterers exhibiting high-quality-factor resonances, such nanoantennas, formed from naturally hyperbolic materials, offer a practical alternative to plasmonic and all-dielectric nanoantennas for realizing ultra-miniaturized photonic devices.

An integrated fiber metasurface device operating in the mid-infrared range has been proposed in [4], demonstrating low-loss transmission as low as 0.03dB/m at 2900nm with excellent single-mode performance. The bilayer metasurface structure excites double Fano resonances, significantly enhancing the quality factor compared to that of a single-layer metasurface. Multipolar analysis reveals that a particular mode can arise from destructive interference between the magnetic dipole and the electric quadrupole. Using the designed parameters, one can achieve a quality factor of 4929, a refractive index sensitivity of 2074 nm/RIU, and a figure of merit of 3050. Furthermore, the quality factor can be optimized by adjusting the structural parameters, as evidenced by their impact on the transmission spectrum. The high quality factor, sensitivity, and figure of merit make this device highly promising for tunable filters, sensing applications, and integrated mid-infrared systems.

Dynamic coding metasurfaces have emerged as versatile platforms for manipulating electromagnetic waves across different frequency ranges. By integrating reconfigurable materials such as vanadium dioxide, these metasurfaces enable real-time control of the reflection, transmission, and scattering properties. Such tunability is particularly valuable for applications in stealth technology, adaptive optics, and programmable holography. The combination of advanced fabrication methods and precise optimization algorithms accelerates the development of metasurfaces for practical, high-performance electromagnetic devices.

The work by Wang et al. [5] reports on a method to develop a terahertz vanadium-dioxide-based dynamic coding metasurface capable of achieving dual-polarized, dual-band, and wide-angle radar cross-section (RCS) reduction. The meta-atom comprised a resonator, a dielectric layer, and a metallic backing. The resonator was constructed from two split-ring resonators embedded with patches of vanadium dioxide. Simulation results verified that tuning the vanadium dioxide state effectively modifies the operating frequency range and Pancharatnam–Berry phase of the meta-atom. Constraining the rotation angles to 0° and 90° was shown to introduce Pancharatnam–Berry phase values of 0° and 180°, respectively, for left- and right-circularly polarized (LCP and RCP) waves. A 1-bit coding metasurface, optimized using the whale optimization algorithm, was designed for validation. Simulation outcomes demonstrated that for vertically incident LCP and RCP waves, switching vanadium dioxide patches between insulating and metallic states achieves RCS reductions exceeding 10 dB at 0.21 THz and 0.39 THz. Moreover, simulations indicated that for oblique incident waves with LCP and RCP and polar angles between 0° and 40°, and azimuth angles ranging from 0° to 360°, the metasurface maintained effective RCS reduction within 0.18–0.24 THz and 0.21–0.39 THz. This approach holds potential for terahertz stealth applications. Additionally, the proposed metasurface could be fabricated using surface micro-electro-mechanical systems, micro-nano machining, and lithographic techniques. Its performance could be validated by far-field pattern testing in a terahertz chamber, while vanadium dioxide state transitions could be induced via heating.

The work by Hayat et al. [6] introduced a microwave absorber based on a flexible metasurface absorber (FMSA) array designed for stealth technologies. The core component features a closed-bracket-shaped design crafted from indium tin oxide (ITO) and printed onto flexible, transparent substrates such as polyvinyl chloride (PVC) and polyethylene terephthalate (PET). A unit cell composed of ITO-PET-PVC was utilized to achieve absorption exceeding 90% across a wide frequency range from 9.85 to 41.76 GHz, attributed to magnetic resonance, with an observed optical transmittance of 74%. Furthermore, after a thorough assessment of the RCS reduction, it was established that the proposed FMSA achieves an RCS reduction that exceeds 10 dB in both flat and conformal configurations, with a relative bandwidth of 124%. The experimental results corroborated the simulations, verifying that the FMSA exhibits both broadband absorption and angular stability. Incorporating flexible substrates, such as PET and PVC, ensures that the designed structure is suitable for microwave stealth applications and can be readily adapted to operate in different frequency ranges.

In conclusion, the research presented in the Special Issue “Advances in Photonic Metasurfaces and Metastructures” emphasizes the transformative potential of metasurfaces in controlling light across various frequencies and applications. Integrating novel materials, dynamic reconfigurability, and precise geometric design has paved the way for the development of compact and multifunctional optical devices. These innovations hold significant promise for diverse fields such as communication, sensing, stealth technology, and imaging, driving the future of photonics and photonic integration.
